# Prediction of response and survival in patients with chronic-phase chronic myeloid leukemia treated with omacetaxine mepesuccinate: logistic regression and landmark analyses

**DOI:** 10.1038/bcj.2015.104

**Published:** 2015-12-11

**Authors:** M Wetzler, H M Kantarjian, F E Nicolini, J H Lipton, L Akard, M Baccarani, H J Khoury, E Li, M Munteanu, J Cortes

**Affiliations:** 1 Roswell Park Cancer Institute, Leukemia Section, Buffalo, NY, USA; 2 Department of Leukemia, Division of Cancer Medicine, The University of Texas MD Anderson Cancer Center, Houston, TX, USA; 3 Department of Hematology, Centre Hospitalier Lyon Sud, Pierre Bénite, France; 4 Princess Margaret Cancer Centre, Allogeneic Blood and Marrow Transplant Program, Toronto, Ontario, Canada; 5 Indiana Blood and Marrow Transplantation, Hematology, Indianapolis, IN, USA; 6 University of Bologna, Institute of Hematology and Medical Oncology, Bologna, Italy; 7 Department of Hematology and Medical Oncology, Emory University School of Medicine, Atlanta, GA, USA; 8 PharmaStat LLC, Newark, CA, USA; 9 Teva Branded Pharmaceutical Products R&D, Clinical Development, Frazer, PA, USA

Although many chronic myeloid leukemia (CML) patients initially do well with tyrosine kinase inhibitors (TKIs), some patients develop resistance or intolerance to multiple TKIs and need further therapy. Omacetaxine mepesuccinate (omacetaxine), a protein synthesis inhibitor, represents a new class of treatment that can produce major cytogenetic response (MCyR) in patients with CML who have developed resistance or intolerance to TKIs. The US Food and Drug Administration approved subcutaneous omacetaxine for treatment of CML in chronic-phase (CP) and accelerated-phase patients, with resistance or intolerance to two or more TKIs based on efficacy analysis of a subset of patients from two phase 2, open-label, international, multicenter studies.^1, 2^ Among the 76 evaluable patients with CML-CP in the efficacy analysis, MCyR was reported in 14 patients (18.4%), including confirmed complete cytogenetic response (CCyR) in six patients (7.9%), with a median MCyR duration of 12.5 months.^2, 3^ Median progression-free survival (PFS) and overall survival (OS) in CML-CP patients were 9.6 months (95% confidence interval (CI) 6.8–11.3 months) and 40.3 months (95% CI 23.8 months–not reached), respectively.

To identify patients with prior TKI resistance or intolerance most likely to benefit from a non-TKI treatment, we evaluated the association between baseline characteristics and achievement of MCyR in *post hoc* analysis of data from all 76 CML-CP patients included in the pivotal efficacy analysis of omacetaxine.^1, 2^ We also examined the association of response with survival via retrospective landmark analyses. The final data cutoff for this analysis was 12 October 2012. Eighteen baseline variables were examined for association with the achievement of MCyR using both univariate analysis (that is, Fisher's exact test) and multivariate analysis (that is, logistic regression; Table 1). Four of 18 baseline variables evaluated in univariate analysis were associated with the increased likelihood of achieving MCyR with omacetaxine at a significance level of *P*⩽0.1; these included achievement of complete hematologic response (CHR) with the most recent TKI; achievement of MCyR with the most recent TKI; no hydroxyurea (HU) use at baseline; and presence of CHR at baseline. The number of prior TKIs was not associated with achievement of MCyR. Among patients with resistance to two or more prior TKIs at baseline, 13 (19%) achieved MCyR with omacetaxine treatment (including seven CCyR and six partial CyR). Two of seven patients (29%) with intolerance to two or more TKIs at baseline achieved MCyR. Mutational status of BCR-ABL1 was also not predictive of response. Additional univariate analyses of MCyR rates by baseline CHR status and HU use showed the highest response rate (5/13 patients, 39%) in patients who were in CHR at baseline without the use of HU, and the lowest rate in patients who were not in CHR at baseline despite the use of HU (2/33 patients, 6%); MCyR rate was 25% (2/8) in patients having CHR at baseline with HU use and 23% (5/22) in patients without CHR and without HU use at baseline.

We further assessed the predictive value of baseline variables for the achievement of MCyR using a logistic regression model. Baseline variables with *P*-values⩽0.35 in the univariate analysis were selected for inclusion and variables that may cause multilinearity were removed from the logistic model. The final model included five baseline variables (baseline CHR, baseline HU use, MCyR to the most recent TKI, number of prior approved TKIs and MCyR to previous imatinib), with an acceptable goodness-of-fit (Akaike information criterion score of 68.22) and an *R*-squared value (*R^2^*)=0.1940 for the logistic analysis. The achievement of MCyR with the most recent TKI (odds ratio 4.951 (95% CI 1.234–19.866); *P*=0.0240) was the only statistically significant predictor for achieving MCyR on omacetaxine of the five factors remaining in the final model (Table 1).

Results from both univariate and logistic regression analyses showed that patients who achieved MCyR to their most recent TKI (before progression) may more likely achieve MCyR with omacetaxine. This is similar to other models in which response to first-line imatinib was a predictor of CCyR to subsequent dasatinib or nilotinib.^4, 5^ Taken together, these results are particularly interesting in that the previous response to initial TKI treatment may predict a subsequent response whether the secondary treatment is a TKI or not. In this trial, 9% of patients without MCyR and 41% of those with MCyR to prior TKI achieved MCyR with omacetaxine (Table 1). The finding that patients without CHR at baseline despite the use of HU were least likely to achieve MCyR with omacetaxine (in univariate analysis) may simply be owing to the presence of more proliferative disease in this patient subset. Importantly, response to omacetaxine is not dependent on BCR-ABL1 mutation status (T315I or other mutations), as would be expected since the activity of omacetaxine is independent of direct BCR-ABL1 binding.^6^ This contrasts with treatment with second-generation TKIs, in which the absence of specific, more sensitive baseline mutations was associated with longer PFS.^7^ Preliminary *post hoc* analyses in patients with CML-CP treated with ponatinib also noted that presence of T315I was not a significant prognostic factor for response.^8^

We also examined the association of response with survival using retrospective landmark analyses to estimate the median OS from time of omacetaxine initiation in patients with/without CHR at 3 months and with/without CyR or MCyR at 3, 6 and 12 months who remained on treatment at the specified time points. Of 76 CML-CP patients treated, 53 (70%) remained on treatment at 3 months, 43 (57%) at 6 months and 25 (33%) at 12 months. The 47 patients who achieved or maintained CHR by 3 months demonstrated a longer median OS than the 6 patients without CHR (49.5 vs 15.0 months; Table 2); median OS was not reached among the 17 patients with CHR at baseline who maintained response at 3 months (95% CI 17.8 months–not reached) and was 40.3 months (95% CI 22.9–59.4 months) in the 30 patients without CHR at baseline who achieved CHR with omacetaxine. A correlation between MCyR at 3 months and 6 months (*n*=8 each) and median OS was not reached (all *P*>0.5), possibly because of the small size of the cohort. For the 6 patients achieving MCyR by 12 months, median OS was not reached and all 6 patients achieved CCyR (five patients were alive with a median follow-up of 48.7 months (range 43.2–57.2 months)), compared with a median OS of 59.4 months in the 19 patients without MCyR at 12 months (*P*=0.3375).

Interpretation of these results is limited by the small cohort, single-arm design and the exploratory, *post hoc* nature of these analyses. However, this information may help inform treatment decisions when considering a non-TKI approach in CML-CP patients.

These results indicate that achievement or maintenance of CHR through 3 months and MCyR at 12 months with omacetaxine may be associated with favorable survival (⩾30 months) in CML-CP patients previously treated with two or more TKIs. Meaningful response milestones may take longer to achieve in heavily pretreated patients receiving omacetaxine and may differ from those used for TKIs. Nevertheless, achievement and/or maintenance of CHR at 3 months with omacetaxine may be a clinically meaningful indicator of benefit to omacetaxine.

## Figures and Tables

**Table 1:** Univariate analysis and multivariate (logistic regression analysis) to examine specific baseline variables with the achievement of MCyR on omacetaxine

*Baseline variable*		*MCyR rate* n*/*N *(%)*	*OR*	*95% CI*	P*-value*
*Univariate analysis of association with MCyR*					
Age	<65 years ⩾65 years	12/53 (23) 2/23 (9)	3.073	0.629–15.017	0.2052
Sex	Male Female	10/47 (21) 4/29 (14)	1.689	0.476–5.989	0.5472
BSA	⩾1.94 kg/m^2^ <1.94 kg/m^2^	9/38 (24) 5/38 (13)	2.048	0.616–6.812	0.3754
Time since CML diagnosis	<75 months ⩾75 months	9/39 (23) 5/37 (14)	1.920	0.578–6.383	0.3782
Time since last TKI dosing	⩾1 month <1 month	10/47 (21) 4/29 (14)	1.689	0.476–5.989	0.5472
Duration of all prior TKI treatment	<4 years ⩾4 years	10/38 (26) 4/38 (11)	3.036	0.859–10.732	0.1373
Number of prior approved TKIs	2 TKIs 3 TKIs	10/40 (25) 4/36 (11)	2.667	0.755–9.420	0.1465
MCyR to previous imatinib	Yes No/unknown	6/19 (32) 8/57 (14)	2.827	0.833–9.599	0.1004
MCyR to the most recent TKI	Yes No/unknown	9/22 (41) 5/54 (9)	6.784	1.939–23.74	0.0026^a^
CHR to the most recent TKI	Yes No/unknown	13/53 (25) 1/23 (4)	7.150	0.876–58.349	0.0520^a^
Baseline HU use	Yes No	4/41 (10) 10/35 (29)	3.7	1.044–13.118	0.0424^a^
Baseline CHR	Positive Negative	7/21 (33) 7/55 (13)	3.429	1.028–11.44	0.0509^a^
T315I mutation	Absent/unknown Present	11/56 (20) 3/20 (15)	1.385	0.344–5.578	0.749
Any mutation	Absent/unknown Present	9/42 (21) 5/34 (15)	1.582	0.476–5.26	0.5578
Baseline blasts	0 to <5% ⩾5% or missing	9/50 (18) 5/26 (19)	0.922	0.274–3.102	1.0
Baseline basophils	0 to <2% ⩾2% or missing	3/27 (11) 11/49 (22)	0.432	0.109–1.708	0.3546
Baseline clonal evolution	Presence Absence	2/12 (17) 12/64 (19)	0.867	0.168–4.48	1.0
Baseline spleen size	0 to <10cm ⩾10 cm or missing	7/39 (18) 7/37 (19)	0.937	0.294–2.991	1.0
					
*Logistic regression analysis*^a^					
Baseline CHR	Positive Negative	7/21 (33) 7/55 (13)	2.831	0.728–11.008	0.1332
Baseline HU use	Yes No	10/35 (29) 4/41 (10)	2.629	0.650–10.624	0.1750
MCyR to the most recent TKI	Yes No/unknown	9/22 (41) 5/54 (9)	4.951	1.234–19.866	0.0240^b^
Number of prior approved TKIs	2 TKIs 3 TKIs	10/40 (25) 4/36 (11)	2.454	0.607–9.922	0.2078
MCyR to previous imatinib	Yes No/unknown	6/19 (32) 8/57 (14)	1.447	0.332–6.300	0.6223

Abbreviations: BSA, body surface area; CHR, complete hematologic response; CI, confidence interval; CML, chronic myeloid leukemia; HU, hydroxyurea; MCyR, major cytogenetic response; OR, odds ratio; TKI, tyrosine kinase inhibitor.

a Baseline variables with *P*-value ⩽0.1 and number of prior approved TKIs were included in the logistic regression model. CHR to the most recent TKI was excluded because of collinearity in logisitic regression analysis.

b *P*-value ⩽0.05.

**Table 2:** Median OS in CML-CP patients by level of response at 3, 6 or 12 months

*Time point, mo*	*Response*	n	*Deaths,* n *(%)*	*Median OS, mo*	*95% CI*
3	CHR	47	21 (45)	49.5	31.6–NR
	No CHR	6	4 (67)	15.0	7.8–NR
	Any CyR	11	4 (36)	49.3	9.6–NR
	No CyR	42	21 (50)	49.5	22.9–NR
	MCyR	8	4 (50)	49.3	9.6–49.3
	No MCyR	45	21 (47)	49.5	22.9–NR
					
6	Any CyR	14	4 (29)	49.3	40.3–NR
	No CyR	29	15 (52)	49.5	20.3–NR
	MCyR	8	4 (50)	49.3	14.2–49.3
	No MCyR	35	15 (43)	59.4	27.8–NR
					
12	Any CyR	12	1 (8)	NR	49.3–NR
	No CyR	13	8 (62)	49.5	17.8–59.4
	MCyR	6^a^	1 (17)	NR	49.3–NR
	No MCyR	19	8 (42)	59.4	27.8–59.4

Abbreviations: CHR, complete hematologic response; CI, confidence interval; CML-CP, chronic-phase chronic myeloid leukemia; CyR, cytogenetic response; MCyR, major cytogenetic response; mo, months; NR, not reached; OS, overall survival.

a All patients with MCyR by 12 months had complete CyR (0% Philadelphia chromosome-positive cells).
